# The impact of Pleistocene glaciations and environmental gradients on the genetic structure of *Embothrium coccineum*

**DOI:** 10.1002/ece3.9474

**Published:** 2022-11-09

**Authors:** Francisco Sepúlveda‐Espinoza, Ariana Bertin‐Benavides, Rodrigo Hasbún, Óscar Toro‐Núñez, Antonio Varas‐Myrik, Diego Alarcón, Marie‐Laure Guillemin

**Affiliations:** ^1^ Laboratorio de Epigenética Vegetal, Departamento de Silvicultura, Facultad de Ciencias Forestales Universidad de Concepción Concepción Chile; ^2^ Facultad de Ciencias, Instituto de Ciencias Ambientales y Evolutivas Universidad Austral de Chile Valdivia Chile; ^3^ ONG Conciencia Sur Concepción Chile; ^4^ Laboratorio de Genómica Forestal, Centro de Biotecnología Universidad de Concepción Concepción Chile; ^5^ Departamento de Botánica, Facultad de Ciencias Naturales y Oceanográficas Universidad de Concepción Concepción Chile; ^6^ Departamento de Ciencias Ecológicas, Instituto de Ecología y Biodiversidad Universidad de Chile Ñuñoa Chile; ^7^ Núcleo Milenio MASH, Instituto de Ciencias Ambientales y Evolutivas, Facultad de Ciencias Universidad Austral de Chile Valdivia Chile; ^8^ IRL 3614 Evolutionary Biology and Ecology of Algae, CNRS Sorbonne Université, Pontificia Universidad Católica de Chile, Universidad Austral de Chile Roscoff France; ^9^ Centro FONDAP de Investigación en Dinámica de Ecosistemas Marinos de Altas Latitudes (IDEAL) Valdivia Chile

**Keywords:** divergence time, isolation by environment, last glacial maxima, population genomics, Proteaceae, temperate forest

## Abstract

The South American temperate forests were subjected to drastic topographic and climatic changes during the Pliocene‐Pleistocene as a consequence of the Andean orogeny and glacial cycles. Such changes are common drivers of genetic structure and adaptation. *Embothrium coccineum* (Proteaceae) is an emblematic tree of the South American temperate forest (around 20°S of latitude) that has strongly been affected by topographic and climatic events. Previous studies have shown a marked genetic structure in this species, and distinct ecotypes have been described. Yet, little is known about their adaptive genetic responses. The main goal of this study was to investigate the effects of historical and contemporary landscape features affecting the genetic diversity and connectivity of *E*. *coccineum* throughout its current natural distribution. Using over 2000 single nucleotide polymorphisms (SNPs), we identified two genetic groups (a Northern and a Central‐Southern group) that diverged around 2.8 million years ago. The level of genetic structure was higher among populations within the Northern genetic group than within the Central‐Southern group. We propose that these differences in genetic structure may be due to differences in the assemblages of pollinators and in the evolutionary histories of the two genetic groups. Moreover, the data displayed a strong pattern of isolation by the environment in *E*. *coccineum*, suggesting that selection could have led to adaptive divergence among localities. We propose that in the Chilean temperate forest, the patterns of genetic variation in *E*. *coccineum* reflect both a Quaternary phylogenetic imprint and signatures of selection as a consequence of a strong environmental gradient.

## Graphical abstract

Using 2155 SNPs we detected two main genetic groups, which have diverged some 2.5 million years ago, in *Embothrium coccineum*. Both glacial cycles and selection to the strong environmental gradients have largely shaped the genetic diversity of this native Chilean tree.

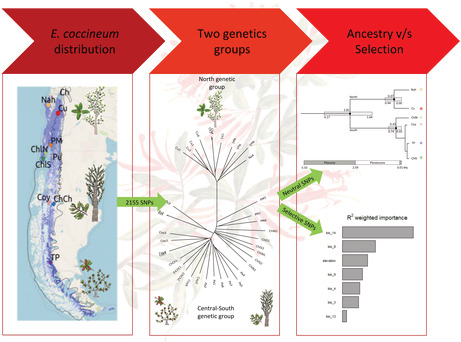

## 1 INTRODUCTION

South American temperate forests are considered of great ecological and evolutionary importance because of their isolation and high degree of endemism (Smith‐Ramírez et al., 2007). In Chile, temperate forests stretch from 35°S down to 55°S. These form a continuous area that can be described as a “biogeographic island” because it is separated from its ancestral sources of biota by nearly impassable barriers such as deserts, mountains, and oceans (Villagrán, 1991). As a result, the climate and topography characterizing the narrow strip (2000 km long and 120 km wide) of Chilean temperate forests comprise a highly heterogeneous landscape of multiple forest and soil types, climatic conditions, and disturbance regimes (Loguercio et al., 2018). Adaptive processes allowing persistence in a mosaic of different habitats may have played an important role in the diversification of plant species in the temperate forests of southern South America (Johnson et al., 2014; Prunier et al., 2017). In the same way, the pattern of genetic divergence within a single species, with an extensive distribution over a strong environmental gradient, can also be marked by selection (Grossiord et al., 2004; Saldana et al., 2007; Zeppel et al., 2014). *Embothrium coccineum*, J.R. Forts. & G. Forst (Dimitri, 1972) is an endemic species of Gondwanan origin with a distribution range that covers the extent of the temperate forest biome throughout Chile and Argentina (Alberdi & Donoso, 2004; Chalcoff et al., 2008; Zegers, 1994). In *Embothrium*, the presence of distinct ecotypes has been linked to differences in water accessibility, and the amount of snow coverage and the distribution of its ecotypes correspond to the genetic structure found using isoenzymatic genetic markers (Souto & Premoli, 2007). This correlation suggests that *E*. *coccineum* populations may be locally adapted to distinct environmental conditions across its distribution range (Bertin‐Benavides et al., 2020; Souto & Premoli, 2007).

In addition to the possible effect of current selective regimes, climate shifts during the Pleistocene have also strongly altered the spatial patterns of genetic variation of many southern South American taxa (Turchetto‐Zolet et al., 2013; Vidal‐Russell et al., 2011). During the Last Glacial Maximum (LGM, 25,000 years Before Present, BP), a large expanse of temperate forest disappeared under thick ice caps between 42°S and 56°S (Rabassa et al., 2005). Glacial maxima rapidly forced most temperate South American species into glacial refugia north of 42°S (Allnutt et al., 1999; Premoli et al., 2000; Sersic et al., 2011). Some, more cold‐tolerant species, such as *E*. *coccineum*, were also able to survive within the regions affected by glaciation, in refugia where the microclimate, the topography, and geothermal activity hindered the formation of ice caps (Allnutt et al., 1999; Premoli et al., 2000; Sersic et al., 2011). These areas may also have been sources for the restoration of genetic diversity after deglaciation (Comps et al., 2001). Lastly, fossils of pollens from cold‐tolerant species confirm their presence at 11,000 years BP in areas covered by ice during the LGM (Fesq‐Martin et al., 2004; Steubing et al., 1983) and genetic studies using chloroplast gene sequences or nuclear isoenzymatic markers (Souto & Premoli, 2007; Vidal‐Russell et al., 2011) further suggest an overall complex glacial–interglacial history in *E*. *coccineum*.

Recent developments in high‐throughput sequencing technologies and population genomics have allowed to distinguish between the influence of historical and ecological processes on the spatial genetic patterns and variation in South American plants and animals (Hasbún et al., 2016; Turchetto‐Zolet et al., 2013; Varas‐Myrik et al., 2022). If divergent natural selection led to genetic differentiation among populations of *E*. *coccineum* along the characteristic environmental gradients of South American temperate forests, a pattern of isolation by the environment (IBE; Wang & Bradburd, 2014) should be expected. In fact, when selection against migration limits gene flow among populations from distinct environments, genetic differentiation increases with environmental differences (Wang & Bradburd, 2014; Wang & Summers, 2010). To test for the existence of IBE, the level of correlation between genetic differentiation and environmental distances should be compared with the correlation between genetic differentiation and geographic distances to assess which one better explains the genetic structuring of the species. The latter correlation is defined as isolation by distance (IBD; Slatkin, 1993) and can be considered as a null model for which genetic differentiation increases with geographical distance given the local accumulation of genetic differences when the dispersion between populations is geographically restricted. In *E*. *coccineum*, gene flow could for instance be limited by constraints in pollen transport linked to the distribution and behavior of pollinators (i.e., more than 20 species, including birds and insects, are reported as pollinators of *E*. *coccineum*; Chalcoff et al., 2008) and/or anemochorous seed dispersal (Rovere & Premoli, 2005).

The main goal of this study was to evaluate the effects of historical and contemporary landscape features affecting the genetic diversity and connectivity of *E*. *coccineum* throughout its natural distribution using single nucleotide polymorphisms (SNPs) obtained by genotyping‐by‐sequencing (GBS). Patterns of genetic structure were evaluated independently using all SNP loci (i.e., neutral and outlier loci) and those only found in genomic regions that may have been subjected to selection (i.e., only outlier loci). We hypothesized that patterns of genetic variation in *E*. *coccineum* will reflect the impact of historical processes (i.e., glacial/interglacial cycles during the Pleistocene) and the impact of contemporary landscape features, including the selective effects of environmental heterogeneity.

## 2 MATERIALS AND METHODS

### 2.1 Samples collection and DNA extraction

**FIGURE 1:**
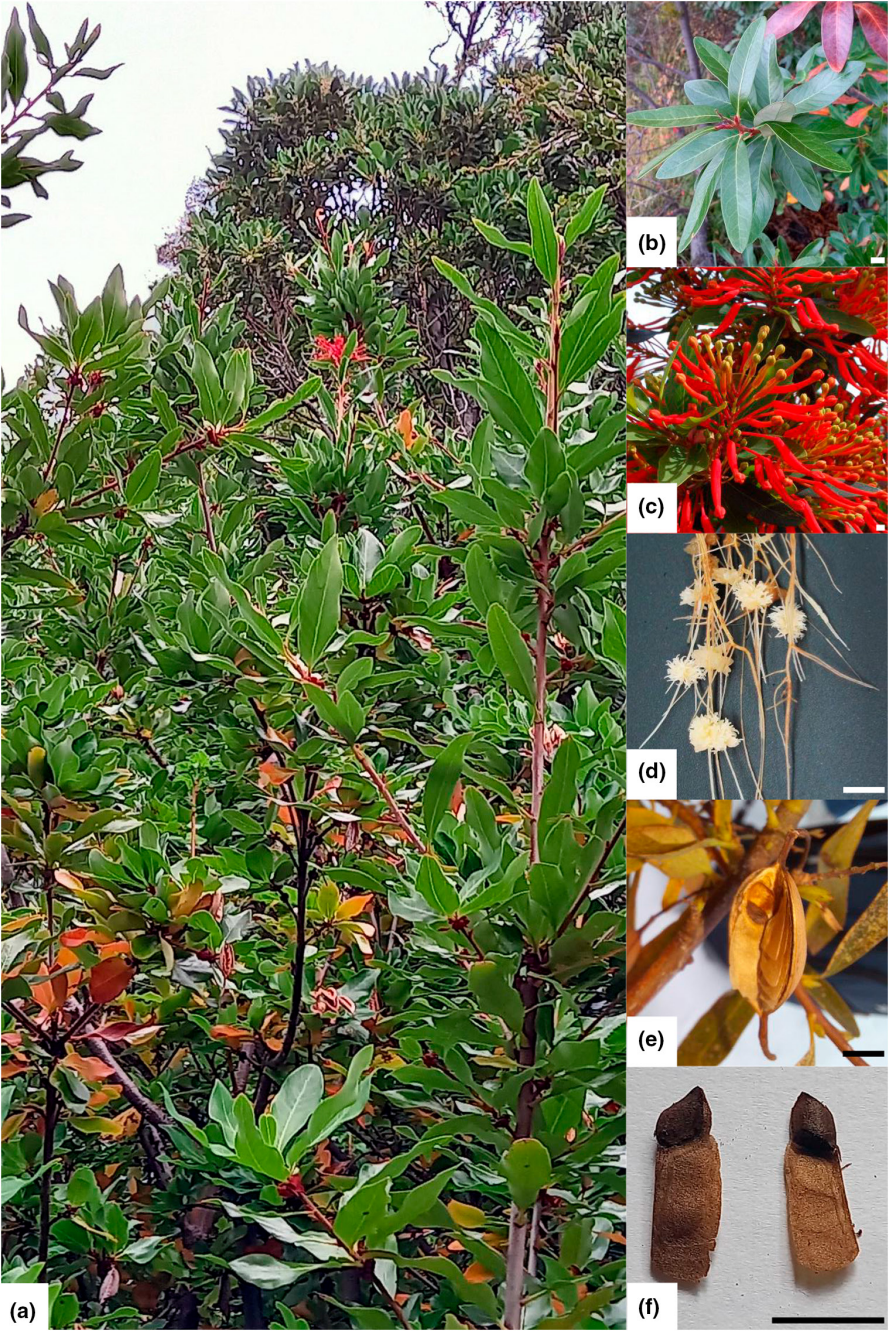
(a) *Embothrium coccineum* adult tree. Inserts are close‐ups of (b) leaves, (c) flowers, (d) cluster roots, (e) follicles, and (f) seeds. Scale bars represent 1 cm. All photographs by Ariana Bertín.

**FIGURE 2:**
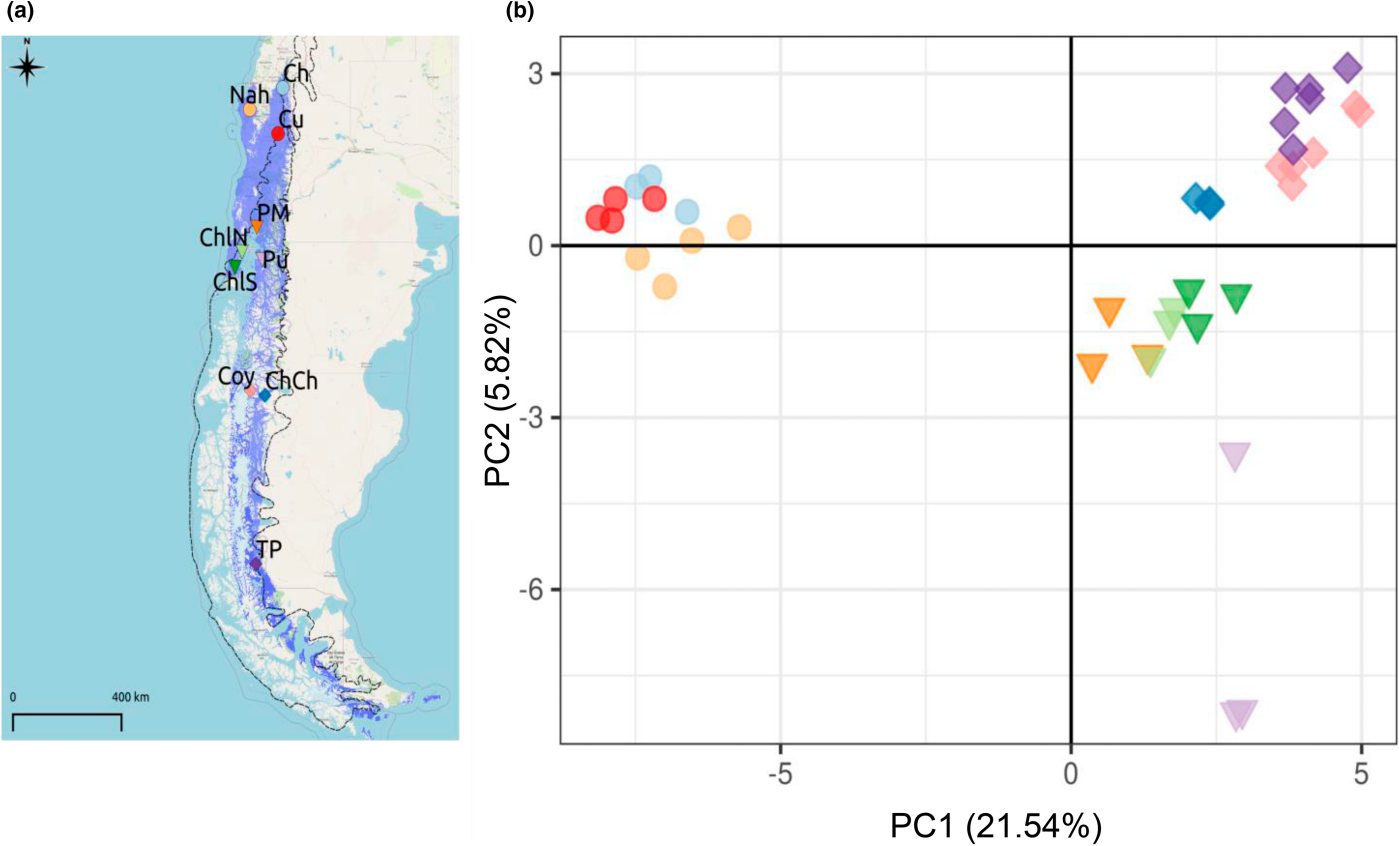
(a) Sampling locations of *Embothrium coccineum*. Circles, triangles, and diamonds represent samples from the northern, central, and southern parts of the species distribution, respectively. *Embothrium coccineum* distribution area is highlighted in light blue and hatched lines represent ice limits from last glacial maxima. (b) Principal component analysis (PCA) based on a 2155 SNP dataset of *Embothrium coccineum* samples. Sampling location codes are as in Table S1. Symbols and colors used for each locality are as in Figure 2a.

Locations were selected to cover an extensive range of climatic conditions across *E*. *coccineum's* (Figure 1) natural distribution. Three locations (Chillán, Nahuelbuta, and Curacautín) represented the northern area of the distribution; four locations (Puerto Montt, Chiloé Norte, Chiloé Sur, and Pumalín) the central area of the distribution; and three locations (Coyhaique, Chile Chico and Torres del Paine) the southern area of the distribution (Figure 2a and Table S1). These three regions are subsequently named Northern, Central, and Southern. Three to six individuals were sampled within each location and five mature leaves were collected per tree. A total of 42 trees were sampled. To calibrate divergence time among the genetic groups of *E*. *coccineum*, two individuals of *Lomatia hirsuta* Diels, a Proteaceae species from the Embothrieae tribe (Sauquet et al., 2009), were included as an outgroup. Mature leaves were transported in a cooler to the laboratory where they were frozen in liquid nitrogen and stored at −80°C until DNA extraction. Genomic DNA was obtained from leaf tissue (50 mg, powdered in a Precellys24 homogenizer; Precellys) using the Qiagen DNeasy Plant kit (Qiagen Inc.) following the manufacturer's instructions. The genomic DNA integrity was evaluated in 1% agarose gels, and its concentration was quantified using a Qubit fluorometer (Invitrogen).

### 2.2 Climatic data collection

The historical values (from the years 1970 to 2000) of 19 environmental variables (i.e., “bio 1” to “bio 19”) and the elevation of each location (Elv; from the package raster v.2.7.15 and the Worldclim data of 2.5 arcmins available at http://worldclim.org/), were used to characterize/define spatial environmental variation. We tested for covariation among environmental variables using the *vifcor*() function from the “usdm” R package (Naimi et al., 2014). Variables presenting no strong covariation (*R* < 0.8; see Fitzpatrick & Keller, 2014) were kept for subsequent analyses (Table S1).

### 2.3 Preparation of the library, high‐throughput sequencing, and genotyping‐by‐sequence (GBS)

Library preparation and high‐throughput sequencing took place at the University of Wisconsin Biotechnology Center (DNA Sequencing Facility). The preparation of the GBS genomic library followed the protocol in Elshire et al. (2011) using the ApeKI restriction enzyme and 44 individual‐specific barcodes. High‐throughput sequencing was performed in an Illumina HiSeq 2000 (Illumina) and single‐strand sequencing runs of 100 bp. The quality of the sequenced raw data was assessed using FastQC (https://www.bioinformatics.babraham.ac.uk/projects/fastqc/) and the raw GBS dataset can be found in the SRA repository (https://www.ncbi.nlm.nih.gov/sra/?term=PRJNA783610).

### 2.4 SNP calling and filtering process

Single nucleotide polymorphism calling was made using the de novo pipeline in Stacks v.2.2 (Catchen et al., 2013). Raw reads from Illumina sequencing were demultiplexed, adapters were removed, and reads with low quality scores were discarded using the process_radtag pipeline in Stacks v.2.2 (Catchen et al., 2013). In order to choose the best fitting assembly parameters and avoid possible biases in the outcome of the genetic structuring of populations due to parameter settings and/or missing data, distinct parameter sets were tested in the *populations* pipeline of Stacks v.2.2 (Catchen et al., 2013). Values of the two main parameters (‐M: the number of mismatches allowed between stacks within individuals; and ‐n: the number of mismatches allowed between stacks between individuals) were chosen following the optimization procedure described in Paris et al. (2017). The minimum minor allele frequency (min‐maf) was set to 0.02 and the maximum observed heterozygosity (max‐obs‐het) to 0.5; while the parameters ‐p (the minimum number of populations where a locus must be present for it to be included) and ‐r (the percentage of individuals in a population that must possess a particular locus for it to be included) were examined using eight combinations comprising an ‐r of 0.6 or 0.7 and a ‐p of 6, 7, 8, or 9. For each combination, the percent (%) of missing data and of heterozygosity, and the number of SNPs were estimated (data not shown). The final data matrix “Population Genetic dataset” (PG_dataset) was built using ‐p 9 and ‐r 0.7, which gave the highest number of SNPs and the lowest percentage of missing data.

An additional dataset was built to estimate the divergence time among the genetic groups of *E*. *coccineum* using a Bayesian multispecies coalescent model (Stange et al., 2018). First, we selected a subset of the northern (using locations Nah and Cu), central (using locations ChlN and ChlS), and the southern (using locations Coy and TP) locations using two individuals per location to maximize the number of SNPs and minimize the amount of missing data. These 12 *E*. *coccineum* individuals were processed jointly with the samples of *L*. *hirsuta*, as an outgroup. A new SNP calling was conducted with the same parameters used for the PG_dataset in the Stacks v.2.2 pipeline (Catchen et al., 2013), resulting in a “divergence time dataset” (DT_dataset). For the latter dataset, optimal values for ‐p and ‐r parameters in order to decrease the percentage of missing data were ‐p 7 and ‐r 0.8. Putative outliers loci were removed from the DT_dataset.

For both datasets (PG_dataset and DT_dataset), only one randomly selected SNP per locus was kept to minimize the probability of coanalyzing linked markers.

### 2.5 Populations' diversity statistics

To explore the distribution of genetic diversity, we calculated in GenoDive v.3.02 the observed (Ho) and expected (He) levels of heterozygosity and the inbreeding coefficient (F_IS_) of each location. The percentage of polymorphic loci and private alleles were estimated using the R package “hierfstat v. 0.04‐22” (Goudet, 2005).

### 2.6 Genetic structure

Four complementary analyses were conducted to characterize the patterns of the genetic structure of *E*. *coccineum* using the PG_dataset. First, we calculated the pairwise FST values among all sampling locations using the function *stamppFst* with 1000 bootstrap replicates across loci to generate *p*‐*values* and a confidence interval of 95% in the R package StAMPP v.1.5.1 (Pembleton et al., 2013). Second, a principal component analysis (PCA) was performed using the *glPCA* function in the R package adegenet v.2.1.1 (Jombart, 2008). Only the first three components were retained to estimate genetic groups. Third, to define the posterior probability of group membership for each individual, we used a model‐based STRUCTURE clustering analysis performed in STRUCTURE v.2.3.4. (Falush et al., 2003). Ten independent simulations allowing admixture were run for each of 10 Ks (from 1 to 10 Ks), with 200,000 Markov chain Monte Carlo replicates (MCMC) and 100,000 samples discarded as burn‐in. STRUCTURE HARVESTER (http://taylor0.biology.ucla.edu/structureHarvester/) was used to determine the optimum K, based on L(K) and ΔK (Earl & VonHoldt, 2012). Lastly, to describe the relationships between individuals and genetic groups, the Nei's genetic distance between individuals was calculated in the R package StAMPP v.1.5.1 (Pembleton et al., 2013). Unrooted neighbor‐joining (NJ) distances' trees were constructed using the individual Nei's distance matrix, as implemented in the R package ape v.5.3 (Paradis & Schliep, 2019).

### 2.7 Outlier detection and association of allele frequency with environmental variables

Two complementary approaches were used to identify candidate loci influenced by selection in the PG_dataset. First, we used a PCA to generate a “model‐free” null distribution of individual genetic distances to detect outlier loci. This analysis was conducted in the R packages PCAdapt v.4.0.3 (Luu et al., 2017) and *q‐*value v.2.16.0 (Storey et al., 2004) with default parameter settings and a search based on K = 10. The detection of outliers was confirmed by plotting the histograms of *p*‐values, and the Mahalanobis (*D* ^2^) test statistic using a False Discovery Rate (FDR) of 5%. Second, we implemented a Bayesian test to detect outlier loci using BAYESCAN 2.1 software (Foll et al., 2008). All simulations were performed using default parameter settings, 20 pilot runs of 5000 iterations followed by 50,000 sampling iterations, and an FDR of 5%. Outlier loci detected by both PCAdapt and BAYESCAN were chosen as candidate adaptive loci.

To search for candidate adaptive loci with allelic frequency influenced by the environmental variation in our study area (see Table S1), a GradientForest (GF) analysis was implemented in the R package gradientForest (Ellis et al., 2012). The GF provides a ranked list of the relative predictive power (*R* ^2^) of all environmental variables allowing for the identification of those that best explain the observed genetic variation. The allele frequencies of candidate adaptive loci (i.e., detected in both PCAdapt and BAYESCAN) were used as the response variable. The GF was fitted using 2000 regression trees per SNP and a variable correlation of 0.5 (Fitzpatrick & Keller, 2014).

### 2.8 Isolation by distance and isolation by environment

To better understand the drivers potentially explaining the observed patterns of spatial genetic variation in *E*. *coccineum*, we performed a distance‐based redundancy analysis (db‐RDA) and a partial db‐RDA at the individual level; first, on the complete PG_dataset and second, only on loci potentially influenced by selection. The RDA is a multiple linear regression method performed between a matrix of dependent variables and a set of matrices for independent variables and has been demonstrated as the more appropriate method (as opposed to for example a Mantel test) when multiple variables are analyzed to identify drivers of population genetic structure (Nadeau et al., 2016; Orsini et al., 2013).

A dependent matrix calculated with a Bray–Curtis dissimilarity index (Bray & Curtis, 1957) was used as a response variable for each individual's co‐dominant variant call format. Predictor matrices were geographic distance (IBD), environmental disparity (IBE), and the degree of shared co‐ancestry (IBA). As environmental data, we used seven environmental variables with a low level of covariation (see Table S1). For the geographic distance matrix, we used Principal Coordinates of Neighborhood Matrices (PCNM) with a truncation threshold of 0.05 for long distances in the function pcnm of the R package vegan v.2.5.7 (Oksanen et al., 2007). PCNM is commonly used to transform spatial distances into rectangular data matrices suitable for constrained ordination or regression analyses (Borcard & Legendre, 2002). Populations' *Q*‐values obtained with STRUCTURE at K = 2 (see Section 3 for details) were used as the co‐ancestry variable. Prior to the analysis, all three independent matrices were scaled to a mean of zero and a variance of one, with the scale function in R (R Core Team, 2020).

Variation among populations of *E*. *coccineum* was partitioned into components explained by geographic distance (IBD), ecological gradients (IBE), co‐ancestry (IBA), or their combination (i.e., constrained by the effects of the remaining two independent matrices), using the vartpart function in the R package vegan v.2.5.7 (Oksanen et al., 2007). The significance of each partition was tested with the anova.cca function and 1000 permutations in the R package vegan v.2.5.7 (Oksanen et al., 2007).

### 2.9 Divergence time among the genetic groups of *Embothrium coccineum*

Using the DT_dataset, we implemented a Bayesian multispecies coalescent model to estimate divergence times among the genetic groups of *E*. *coccineum* (Rannala & Yang, 2003). This analysis was conducted with the coalescent simulator SNAPP v.1.3.0 package (Bryant et al., 2012) of BEAST v.2.4.5 software (Bouckaert et al., 2014). We followed a model accommodated for genome‐wide SNP data, which implements a strict molecular clock and nodes calibrated with biogeographic constraints, resulting from one or more divergence events (Stange et al., 2018). This approach assumes an asymmetric substitution model and equal frequencies. It allows a single parameter to be the rate of the strict molecular clock and is currently the only model supported by SNAPP for this type of data (Stange et al., 2018).

Save a few exceptions, all parameters were set following the Github tutorial “Divergence‐time estimation with SNAPP” (https://github.com/mmatschiner/tutorials/tree/master/divergence_time_estimation_with_snp_data). We used a lognormal distribution for the crown age of the Embothrieae tribe, which was estimated at 66 million years (Myr; Mean = 66.6, Standard Deviation = 0.13, Mean In Real Space = TRUE) using a phylogeny of Proteaceae that included all the genera currently recognized in the family (Sauquet et al., 2009). This estimate was used to approximate the node age of the common ancestor of *E*. *coccineum* in our dataset, which was derived from the sister relationship to the *Lomatia* samples. Additionally, a generation time of 10 years per individual was chosen, following experimental greenhouse observations (Bertin‐Benavides A., personal observation). Alpha and beta parameters were left with a uniform prior of an arbitrary value of 1.0 (Stange et al., 2018).

Species' trees were inferred directly with no prior grouping assignment. We used SNAPP with four independent runs of 2000,000 generations, sampling every 200 generations. The results (i.e., Effective Sample Size, ESS > 300) were checked for mixing all parameters with Tracer v1.7 (Rambaut et al., 2018) and for convergence of split frequencies among runs (Figure S2) with the R package rwty (Warren, 2019). The final trees from each run (corresponding to the 95% highest posterior density [HPD] after discarding a 10% burn‐in of the tree topologies) were combined and subsequently summarized using a maximum credibility tree with TreeSetAnalyser v.2.4.5. The final species' tree was visualized as a cloudgram with DensiTree v.2.2.7 (Bouckaert, 2010).

## 3 RESULTS

In order to evaluate the population structure, the divergence time among genetic groups, and the local adaptation in *E*. *coccineum*, 38 trees were genotyped using GBS (4 of 42 individuals were removed due to the low number of retained reads). A total of 116,606,122 reads were generated and de novo assembled to 1,303,750 loci, from which 473,461 SNPs were called. The application of filtering criteria resulted in a final PG_dataset of 2155 SNPs. For the DT_dataset, comprising 12 individuals of *E*. *coccineum* and two individuals of *L*. *hirsuta*, a total of 320 SNPs were called.

### 3.1 Genetic diversity

The expected and observed heterozygosity (He and Ho, respectively), the inbreeding coefficient (F_IS_), the percentages of polymorphic loci (PPL), and the number of private alleles (PA) are given in Table 1, for each sampling location. Except for the low value (under 15%) observed in Chile Chico (ChCh; PPL = 12.668%), PPL values were broadly homogeneous throughout the species range, ranging from 18.794% in Chillán (Ch) to 42.367% in Coyhaique (Coy; Table 1). The lowest PAs (PA ≤ 30) were found in the four sampling locations: Puerto Montt (PM), Chiloé Norte (ChlN), Chiloé Sur (ChlS), and Chile Chico (ChCh), while the rest ranged from 44 to 83. Pumalín (Pu) and Ch were the only two locations with a negative F_IS_ value; and of those with positive F_IS_ values, four locations (i.e., PM, ChlN, ChlS, and ChCh) had F_IS_ superior to 0.1.

**TABLE 1:** Genetic diversity of *Embothrium coccineum*

ID	*n*	He	Ho	F_IS_	PPL (%)	PA
Ch	3	0.1	0.105	−0.049	18.794	51
Nah	4	0.138	0.132	0.037	32.715	65
Cu	4	0.128	0.126	0.012	30.998	73
PM	3	0.152	0.135	0.114	29.791	30
ChlN	3	0.163	0.139	0.15	36.241	29
ChlS	3	0.152	0.128	0.155	33.132	29
Pu	3	0.134	0.154	−0.148	27.425	83
Coy	6	0.143	0.137	0.043	42.367	62
ChCh	3	0.124	0.106	0.146	12.668	10
TP	6	0.139	0.127	0.086	42.088	44

Abbreviations: F_IS_, inbreeding coefficient; He, expected heterozygosity; Ho, observed heterozygosity; ID, location (as in Table S1); *n*, number of genotyped individuals; PA, private alleles; PPL, percentage of polymorphic loci.

### 3.2 Patterns of population genetic differentiation

The highest pairwise FST values were observed between locations from the northern and southern regions (0.319 < FST < 0.424), followed by those calculated between locations from southern and central regions (0.222 < FST < 0.429), and then by the ones calculated between locations from Central and Southern regions (0.070 < FST < 0.193). The levels of divergence between locations within the same region were generally lower (northern: 0.089 < FST < 0.174; central 0.010 < FST < 0.152; southern: 0.043 < FST < 0.061; Table 2). All pairwise FST values were significant (*p* < .05).

**TABLE 2:** Average FST values for pairwise comparisons among sampling locations of *Embothrium coccineum* in Chile

	Ch	Cu	Nah	PM	ChlN	ChlS	Pu	Coy	ChCh	TP
Ch	–									
Cu	0.163	–								
Nah	0.174	0.089	–							
PM	0.312	0.263	0.222	–						
ChlN	0.309	0.260	0.225	0.024	–					
ChlS	0.345	0.292	0.257	0.041	0.010	–				
Pu	0.429	0.369	0.338	0.152	0.126	0.135	–			
Coy	0.382	0.340	0.319	0.117	0.083	0.071	0.168	–		
ChCh	0.424	0.362	0.341	0.133	0.089	0.070	0.193	0.061	–	
TP	0.384	0.345	0.322	0.133	0.094	0.085	0.185	0.061	0.043	–

*Note*: Higher pairwise FST values represent more divergent localities. All *p*‐values were significant (*p* < .05). Location id as in Table S1.

The PCA showed that *E*. *coccineum* was divided into two major genetic groups: the Northern and the Central‐Southern group (Figure 2b). The northern locations were separated from those corresponding to the Central‐Southern group along the first axis of the PCA (PC1, representing 21.54% of the genetic variance). In contrast, samples from the central and the southern regions were separated along the second axis of the PCA (PC2, representing 5.82% of the genetic variance).

**FIGURE 3:**
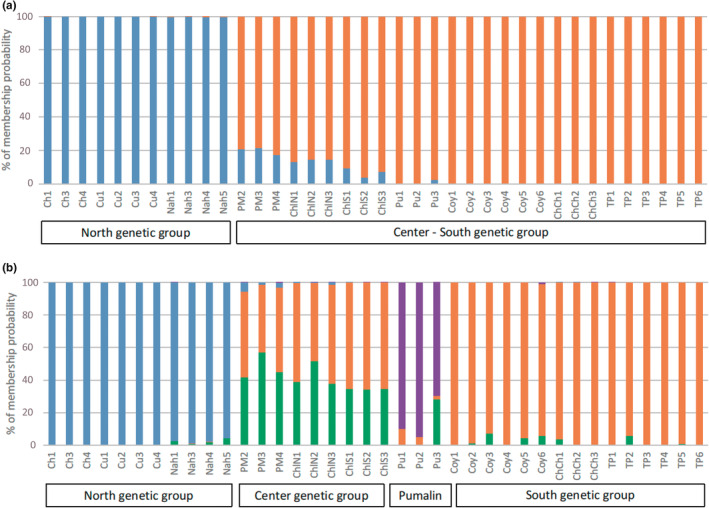
Structure analyses, for K = 2 (a) and K = 4 (b) of 38 *Embothrium coccineum* individuals based on a 2155 SNP dataset. Each individual is displayed from north to south and each individual is represented by a vertical bar. Sampling location codes are as in Table S1. Color corresponds to genetic group, and the proportion of the genome assigned to each genetic group is given along *Y*‐axis.

Results from the model‐based Bayesian clustering approach suggested K = 2 as optimal (i.e., with highest LnP[D] and ΔK) and K = 4 as a possible secondary level of substructure (Figure S1a,b). The STRUCTURE clustering (for K = 2) assigned all individuals from the northern locations to one genetic cluster (in blue) and all other individuals from the central and the southern locations to another genetic cluster (in orange; Figure 3a). The individuals from the northern and southern locations barely displayed signs of admixture; while the individuals from the central region (i.e., from PM, ChlN, and ChlS) had most of their genotype assigned to the Southern genetic cluster (in orange), but with some portion also belonging to the Northern cluster (in blue). The latter is indicative of low levels of admixture in the central region. This level of admixture, however, seemed to recede with latitude (i.e., from 20% of the genome assigned to the Northern genetic cluster (in blue) in PM to less than 10% in ChlS; Figure 3a). The STRUCTURE clustering using K = 4 divided the Central‐Southern genetic group into three new clusters (Figure 3b), while the locations of the northern region remained undifferentiated (as in K = 2). Hence, individuals from the southern region were assigned to one genetic cluster (in orange), while another cluster (in purple) mainly comprised individuals from Pumalín (PU, Central), and the rest of the individuals from the central region (i.e., PM, ChlN, and ChlS) were admixed between the Southern cluster (in orange) and a new, third cluster (in green; Figure 3b).

**FIGURE 4:**
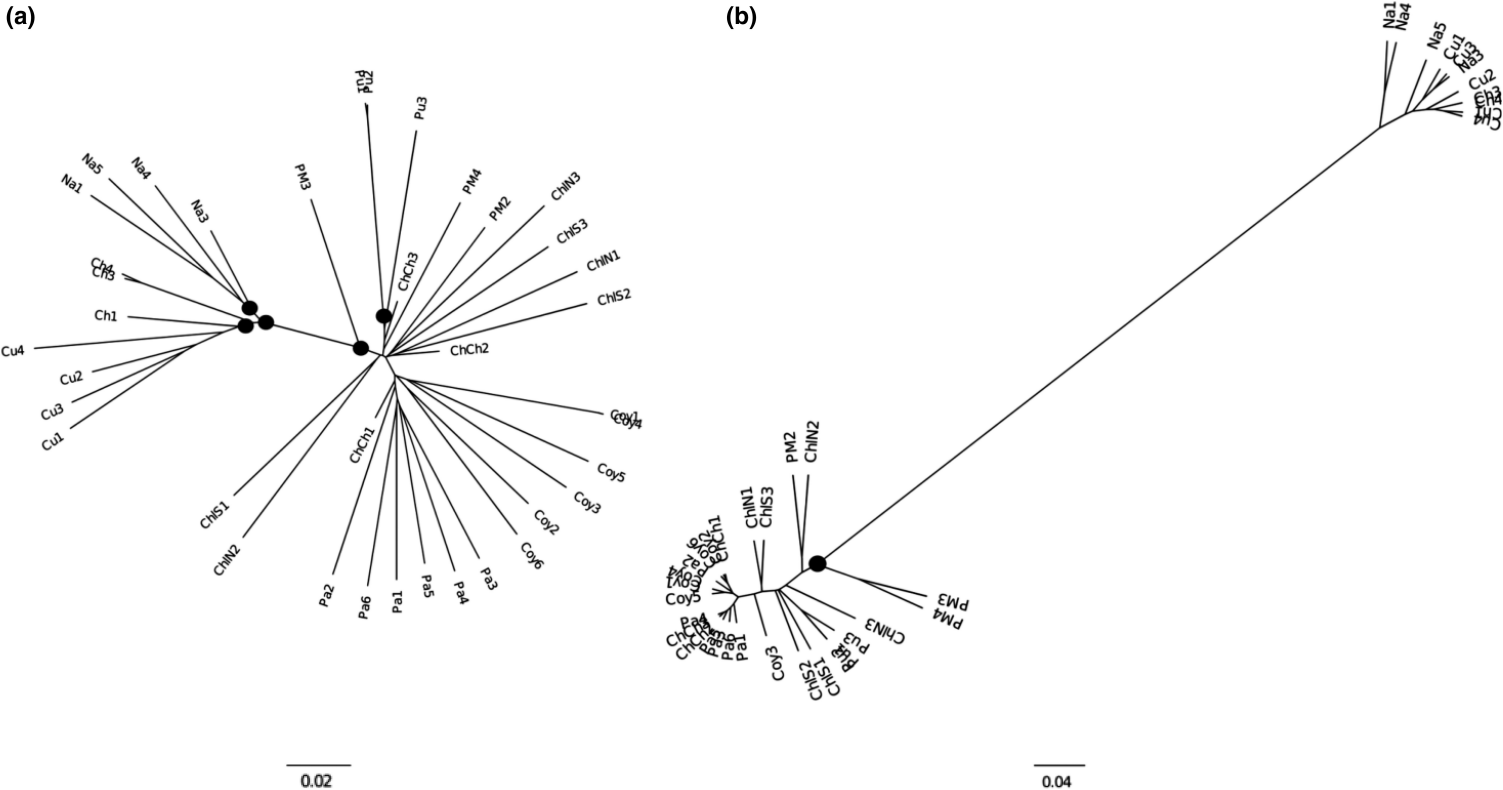
NJ tree reconstructed based on Nei's distance between *Embothrium coccineum* individuals. NJ reconstruction based on (a) the PG_dataset of 2155 SNPs, and (b) the 59 outlier loci putatively under selection. Sampling location codes are as in Table S1. Nodes with high support (>0.95) are filled in black.

The dendrogram reconstructed with Nei's distance tree using the PG_dataset allowed to separate the Northern and Central‐Southern samples (Figure 4a), which was in line with the PCA results and the STRUCTURE clustering analysis. The dendrogram built using only the 59 outlier loci putatively under selection (see below for more details) revealed a much deeper separation between the Northern and Central‐Southern samples, but most subclustering within the major genetic groups was lost (Figure 4b).

### 3.3 Genome scan for outlier loci detection

Within the 2155 loci included in the PG_dataset, PCAdapt detected 132 outlier loci and BAYESCAN detected 89 outlier loci. The two methods concurred in the detection of 59 loci (2.73%), which were kept and designated as potentially adaptive loci in *E*. *coccineum*.

**FIGURE 5:**
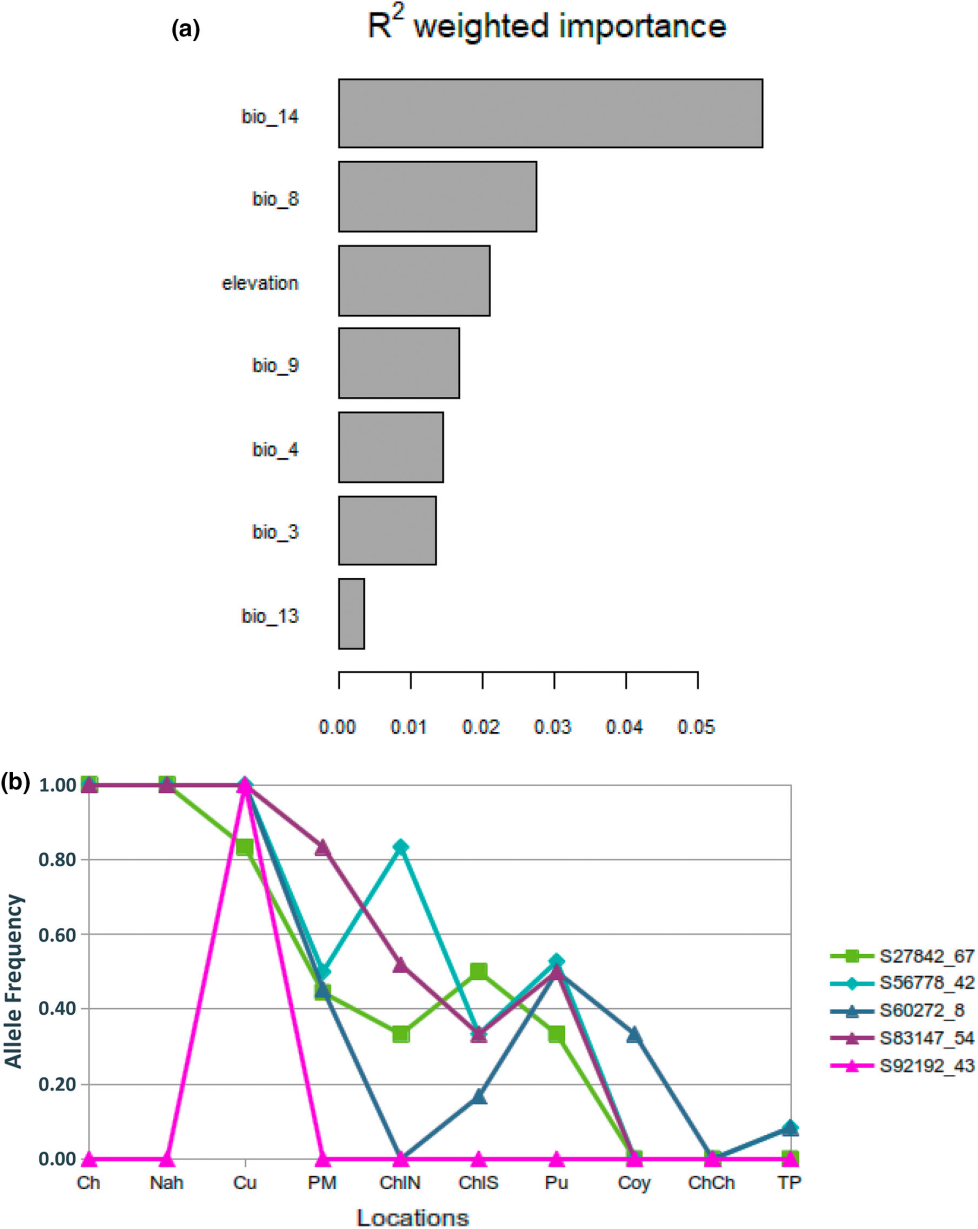
Predictor overall importance plot and change in allelic frequency of five outliers loci with latitude. (a) The *R* ^2^ weighted importance of environmental variables with the low level of covariance explaining the genetic variation across sampling locations as obtained from GF analysis and (b) change in allele frequency between sampling localities, organized from north to south, for the five potentially adaptive loci (i.e, loci: S92192_43, S60272_8, S27842_67, S56778_42, and S83147_54) showing major response to environmental variables. Environmental variables used in the gradientForest analyses are the same as in Table S1; bio 14, precipitation of the driest month (mm); bio 8, mean temperature of the wettest quarter (°C); elevation, elevation (masl); bio 9, mean temperature of the driest quarter (°C); bio 4, temperature seasonality (°C); bio 3, Isothermality (%); bio 13, precipitation of the wettest month (mm).

The GF analysis revealed four environmental factors to have the highest impact (i.e., the highest values of *R* ^2^) on *E*. *coccineum* populations, and comprised precipitation of the driest month (bio 14), mean temperature of the wettest quarter (bio 8), elevation (Elv), and mean temperature of the driest quarter (bio 9; Figure 5a). The *R* ^2^ value of the precipitation of the driest month (*R* ^2^ > .05) was at least two times higher than those among the other three variables of the highest impact.

In addition, GF also produced individual turnover functions for each potentially adaptive loci having an *R* ^2^ > 0 (Fitzpatrick & Keller, 2014). This allowed listing the potentially adaptive loci and the highest correlation of allelic frequencies with each of the four environmental factors of the highest impact on *E*. *coccineum*. The results revealed that the allelic frequencies of two outliers (S92192_43 and S60272_8) strongly correlated with the factor precipitation of the driest month (bio 14; Figure S3a: pink and blue lines, respectively). The allelic frequency of one of those outliers (S60272_8) also exhibited a weak response to the mean temperature of the driest quarter (bio 9; Figure S3d). Three other outliers (S27842_67, S56778_42, and S83147_54) exhibited a significant and concurrent response to the mean temperature of the wettest quarter (bio 8; Figure S3b: green, calypso, and purple lines, respectively); and the allelic frequency of two of those outliers (S272842_67 and S56778_42) also showed a weak response to elevation and mean temperature of the wettest quarter (bio 9; Figure S3c,d; light green and light blue lines, respectively).

Furthermore, the allelic frequencies of four of the five aforementioned outliers (i.e., S60272_8, S27842_67, S56778_42, and S83147_54) gradually changed with latitude (Figure 5b); while the allelic frequencies among locations sampled in the northern region (i.e., Ch, Nah, and Cu) were broadly similar. The remaining locus (S92192_43) had a fixed private allele in the Curacautín (Cu) location. We retrieved the sequences of these outlier loci using the population function of STACKs v2.2 to blast them against the NCBI (www.ncbi.nlm.nih.gov) database using BLASTn, but no significant alignments were found (data not shown).

### 3.4 Isolation by distance and isolation by environment

The full RDA model using the PG_datset explained 59.4% of the total genetic variation and supported the idea of a strong environmental (Env), geographic (Geo), and co‐ancestry (Anc) influence on the allelic variation (Env: adjusted *R* ^2^ = .420, *p* < .001; Geo: adjusted *R* ^2^ = .226, *p* < .001; Anc: adjusted *R* ^2^ = .105, *p* < .001; Table 3). With the partial db_RDA, the contribution of the environmental variables to genetic divergence was much higher than the contribution of the geographic distance or the co‐ancestry (Env | Geo + Anc: adjusted *R* ^2^ = .288; Geo | Env + Anc: adjusted *R* ^2^ = .118; Anc | Env + Geo: adjusted *R* ^2^ = .011; Table 3). In total, 0.178 of the explained variation was confounded between the effects of IBE, IBD, and IBA; 0.405 of the variation remained unexplained (Table 3). Similar results were obtained for the subset of 59 outlier loci (potentially under selection). The partial db‐RDA highlighted the important contribution of environmental variables to the potential local adaptation of *E*. *coccineum* (Env | Geo + Anc: adjusted *R* ^2^ = .244; Table 3).

**TABLE 3:** Redundancy analysis (RDA) partitioning among population genetic variation in *Embothrium coccineum* into three components: Environmental (Env), geographic (Geo), and northern/central‐southern co‐ancestry (Anc).

Combined fractions	All loci	Outlier loci	Individual fractions	All loci	Outlier loci
	Adjusted *R* ^2^	Adjusted *R* ^2^		Adjusted *R* ^2^	Adjusted *R* ^2^
Env	.420	.569	Env | Geo + Anc	.288	.244
Geo	.226	.378	Geo | Env + Anc	.118	.111
Anc	.105	.259	Anc | Env + Geo	.011	.002
Env + Geo	.582	.719	Total confounded	.178	.360
Env + Anc	.476	.606	Unexplained	.405	.283
Geo + Anc	.305	.473			
Env + Geo + Anc	.594	.717			

*Note*: All *p‐*values < .001.

### 3.5 Divergence time

**FIGURE 6:**
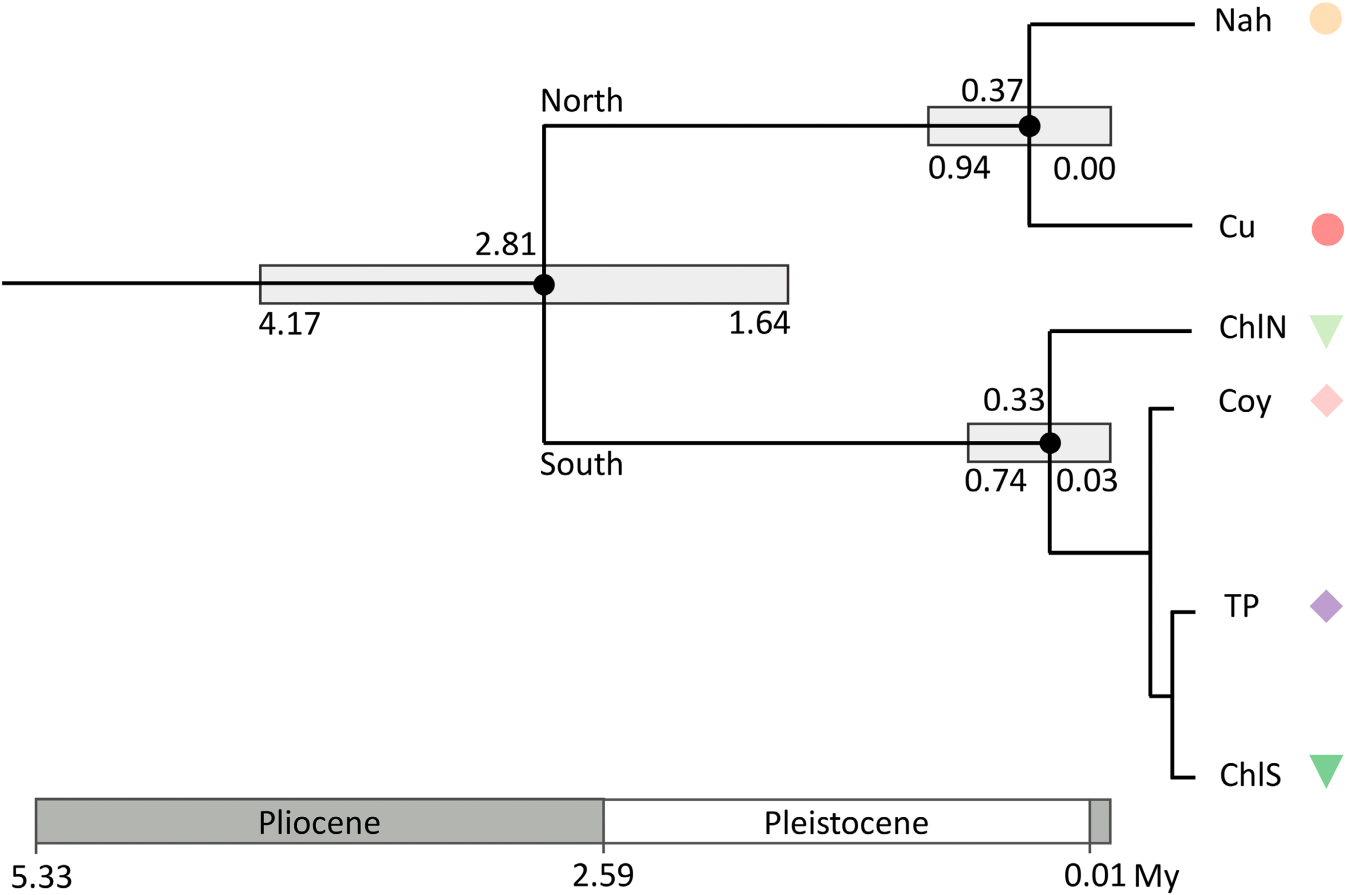
Estimation of divergence time in *Embothrium coccineum* based on Bayesian coalescent analysis using SNAPP. Nodes with high support (posterior probability > .99) are filled in black. Median ages are provided above nodes with 95% highest posterior densities (HDP) below. The divergence time was inferred only for the nodes showing high support (posterior probability > .99). Sampling location codes are as in Table S1; colored symbols for each sampling location match Figure 2a.

The divergence time between the Northern and Central‐Southern genetic groups was estimated at 2.81 Myr (1.64–4.17 Myr), near the end of the Pliocene and at the beginning of the Pleistocene. The locations within the Northern genetic group (Nah and Cu) or within the Central‐Southern genetic group (ChlN and Coy, TP, and ChlS) seem to have diverged much more recently, toward the end of the Pleistocene (0.33/0.37 Myr ago; Figure 6).

## 4 DISCUSSION

In the present study, samples of *E*. *coccineum* from across its distribution range along the Chilean temperate forest were genotyped through GBS to investigate patterns of genetic variation and their possible drivers. The thousands of loci successfully genotyped provided a better resolution and new knowledge on the genetic population structure of *E*. *coccineum* compared with previous studies (Mathiasen et al., 2007; Souto & Premoli, 2007; Souto & Smouse, 2014; Vidal‐Russell et al., 2011). Our data confirmed the existence of two main lineages, a Northern and a Central‐Southern. These genetic groups were already reported in Souto and Premoli (2007), but the GBS dataset was able to reveal fine‐scale genetic differentiation, especially within the Northern lineage. Inference of divergence time between the two genetic groups suggested that intraspecific divergence occurred around 2.8 Myr ago, at the beginning of the Pleistocene, driven by climatic changes linked to glacial cycles. Moreover, the heterogeneous environment characterizing the South American temperate forests has been recognized as a significant driver that explains the current genetic makeup of various plants and animals (Premoli et al., 2000). This heterogeneity could also have led to local adaptive divergence in *E*. *coccineum*. Indeed, the strong IBE pattern we detected in *E*. *coccineum* could reflect the impact of a gradient in temperature, precipitation, and elevation (which characterizes the species' distribution range) on its genetic diversity.

### 4.1 Genetic differentiation within *Embothrium coccineum:* two genetic groups separated since the early Pleistocene

Our results confirm the presence of two genetic groups in *E*. *coccineum*, one in the Northern and one in the Central‐Southern part of the species distribution. These results support previous studies, yet at different levels of spatial resolution (Souto & Premoli, 2007; Vidal‐Russell et al., 2011). The alloenzyme study, for instance, revealed only a weak geographic structure, where some northern and southern locations clustered together (Vidal‐Russell et al., 2011). The authors explained this pattern by proposing a possible effect of glacial survival in multiple refugia followed by recolonization and/or the existence of actual gene flow blurring the trace of the historical geographic structure. Contrastingly, the other study based on chloroplast genetic markers revealed a distinct southern group characterized by a very low genetic variation. In the latter, two hypotheses were used to explain this pattern: (1) the existence of northern and southern glacial refugia as well as a recent colonization event from the southern to the northern part of the distribution of the species, or (2) a unique refugium in the north and a recent recolonization toward the south (Souto & Premoli, 2007).

The genomic SNP data, given their high resolution, were unambiguously able to confirm the idea of the two proposed genetic groups and further evidenced a very high genetic similarity among individuals from the central and southern regions. The divergence time estimated among locations within the Northern lineage with the genomic SNP data was much more recent (e.g., some 0.35 Myr ago) than the divergence between the two main lineages. The divergence between Northern and Central‐Southern lineages was estimated at 2.8 Myr ago, which corresponds to the Late Pliocene and Early Pleistocene. Herewith, our study provides a refined picture of the possible divergence time between the main genetic clusters of *E*. *coccineum* but also allows for an attempt on defining mechanisms that generated the patterns of genetic structure observed at a regional and a local scale. However, the low number of localities sampled and large gaps in the species distribution not covered by our study limit our capacity to locate the boundaries between the North and Center‐South genetic groups in *E*. *coccineum*. Another potential limitation of our study is the low number of individuals sequenced within each population. To take this problem into account, we mostly developed analyses based on differences among individuals (e.g., PCA, STRUCTURE, and unbiased genetic distance between genotypes). Still, it has been shown that consistent FST values can be estimated between populations constituted by only two individuals when a high number of SNP markers (>1500 loci) are used (Nazareno et al., 2017).

Our results revealed the role of Pleistocene glacial cycles as contributors to the genetic architecture of the species and associated environmental changes as possible drivers of divergence in *E*. *coccineum*. In line with our conclusions, the impact of glacial/interglacial contractions and expansions has already been reported on the genetic makeup of South American terrestrial temperate species of herbaceous plants and animals (e.g., plants: *Araucaria araucana*, *Austrocedrus chilensis*, *Calceolaria polyrhiza*, *Fitzroya cupressoides*, *Hordeum comosum*; animals: *Abrothrix longipilis*, *Calomys musculinus*, *Dromiciops gliroides*, *Euneomys chinchilloides*, *Liolaemus buergeri*; See Sersic et al., 2011 for more details).

Southern South American forests are composed of paleo‐flora with variable origins (Segovia et al., 2013; Villagrán & Hinojosa, 1997). Fossil records indicate that during the early Paleocene, this region supported a widespread tropical flora (Hinojosa & Villagrán, 1997). During the Oligocene‐Miocene transition, in response to colder and drier conditions linked to the opening of the Drake Passage, the creation of the Antarctic Circumpolar Current and the steep increase in Antarctic ice caps, a diverse mixed paleo‐flora was established that included Godwanan species preadapted to cool temperate conditions (Poole et al., 2003; Zachos et al., 2001). Nonetheless, the Pleistocene glacial cycles have dramatically altered the landscape of southern South America (Martínez & Kutschker, 2011; Ponce et al., 2011; Ramos & Ghiglione, 2008) and impacted the composition of the local biota (Rabassa et al., 2011). Currently, only one‐third of the woody genera in southern South America are related to ancient paleo‐flora that occupied southern South America in pre‐Quaternary times (Hinojosa et al., 2006; Villagrán & Hinojosa, 1997; Wardle et al., 2001). Palynological records and comparative phylogeographic studies in southern South America propose the existence of various areas where terrestrial plants and animals survived during glacial maxima of the Quaternary. Various studies (e.g., Markgraf, 1983; Vidal et al., 2005) propose the existence of refugia located north of the area heavily impacted by ice (e.g., ranging between 36°S–40°S and 54°S). For example, the absence of pollen records at 41°S (Heusser et al., 1999; Moreno, 1997; Moreno et al., 1999; Moreno & León, 2003) and in Chiloé (Abarzúa et al., 2004; Heusser & Heusser, 2006; Villagrán, 1988) of some native plants such as *Eucryphia codifolia* and *Caldcluvia paniculata* during the LGM suggests a contraction of the ice‐sheet line to the north during glacial maxima. However, Sersic et al. (2011) reported genetic patterns strongly supporting the existence of in situ refugia south of 42°S in plants and animals. Indeed, in these areas, including some parts of the southern Patagonian Steppe, a higher genetic diversity has been observed compared with areas where only postglacial colonization events took place, indicating a high probability of such in situ refugia (Hewitt & Ibrahim, 2001; Huck et al., 2009; Pinceel et al., 2005). In the case of *E*. *coccineum*, information based on cpDNA haplotypes suggested that southern populations had been affected by glacial cycles, declining gradually in size since the Pleistocene (100 ky–12 ky ago) and then rapidly increasing after the LGM (Vidal‐Russell et al., 2011). We found relatively high genetic diversity and polymorphic loci in Nahuelbuta, Curacautín, Chiloe Norte, Coyhaique, and Torres del Paine, and the number of private alleles was also high in these locations. Combined with the observed pattern of genetic structure, these results support the hypothesis of *E*. *coccineum* surviving glacial periods of the Pleistocene in various refugia, including at least two refugia located south of 42°S. The existence of in situ southern refugia in *E*. *coccineum* is also supported by fossil records of cold‐tolerant plant species characteristic of the austral forest reported south of 42°S (Markgraf, 1995). High levels of population divergence and phylogeographic structure attributed to isolation in multiple refugia have been detected in other cold‐tolerant native trees such as *Fitzroya cupressoides* (Allnutt et al., 1999; Premoli et al., 2000, 2003), *Pilgerodendron uviferum* (Ausin et al., 2004; Premoli et al., 2001, 2002), *Araucaria araucana* (Bekessy et al., 2002), *Podocarpus nubigena* (Quiroga & Premoli, 2010), *Nothofagus alpina*, and *Nothofagus pumilio* (Mathiasen & Premoli, 2010; Premoli, 2004; Premoli et al., 2010).

### 4.2 Possible regional differences in pollen and seed dispersal: their effect on gene flow in *Embothrium coccineum*

Our analysis of the genetic differentiation drivers in *E*. *coccineum* identified a significant pattern of isolation by distance (IBD). The existence of IBD indicates that gene flow is limited as geographic distance increases (Wright, 1943). In plants such as *Dactylis glomerata* (Sun et al., 2017) and *Protea rapens* (Prunier et al., 2017), IBD patterns were detected at similar geographic scale, pointing out opportunities for increased allelic exchange between neighboring populations; and indeed some level of connectivity was also observed among *E*. *coccineum* from nearby populations, mainly in the central and southern regions. Contrastingly, STRUCTURE, PCA, and NJ distance tree analyses, as well as the estimation of pairwise FST values, showed that populations from the Northern genetic group of *E*. *coccineum* were highly structured. Therefore, we hypothesize that the population structure differences between localities within the Northern versus the Central‐Southern genetic groups could be, in part, linked to the existence of a more restricted gene flow in the northern part of the distribution compared with the gene flow among central‐southern populations.

Plant gene flow may occur via pollen and/or seed dispersal and is directly affected by the species' reproductive system (Martins, 1987). Seed dispersal decreases competition in areas close to the parent plant and allows the colonization of new sites (Rovere & Premoli, 2005). It also influences the genetic structure of populations since seed transport favors gene flow between and within populations (Hamrick & Nason, 1996), while it restricts potential intraspecific differentiation of ecotypes and subpopulations (Willson et al., 1994). The seeds of *E*. *coccineum* are dispersed by wind and the density of dispersed seeds declines with distance from the tree, for which 95% of the seeds disperse only 5 m from the mother tree (and up to a maximum of 20 m; Rovere & Premoli, 2005). These short dispersal distances could have significant genetic consequences, as the local establishment of propagules could result in a clustering of genetically related individuals, generating a marked genetic structure (Rovere & Premoli, 2005).

In *E*. *coccineum*, gene flow is highly dependent on pollinating agents (Mathiasen, 2004; Rovere & Chalcoff, 2010), and its flowers are visited by more than 20 species, including birds of the Orders Passeriformes and Apodiformes, and insects of the orders Hymenoptera, Diptera, Lepidoptera and Coleoptera (Chalcoff, 2008). The composition of the pollinator assemblage affects pollen transport and seed production. In regions with fewer *E*. *coccineum* pollinators, pollen limitation can be high, leading to low reproductive efficiency (i.e., few fruits produced in relation to the available flowers; Chalcoff, 2008). This could translate into populations presenting reduced genetic diversity and high genetic structure due to the lack of genetic exchange (Rovere & Chalcoff, 2010). Pollinating insects, generally characterized by low migration capacities, are distributed throughout the distribution range of *E*. *coccineum*, even if they are more abundant and visit the plants more frequently in populations located in drier and sunnier climates (i.e., northern locations; Rovere & Chalcoff, 2010). Contrastingly, birds, such as the hummingbird *Sephanoides sephaniodes*, are abundant in the southern region, characterized by the coldest and most humid conditions where they act as one of the principal pollinators (Chalcoff, 2008). We propose that differences in the distribution and behavior of the pollinators between northern and central‐southern regions could lead to the observed genetic structure in *E*. *coccineum*, with a lower dispersal capacity in the northern locations compared with the central‐southern ones, leading to a higher level of structure in the former.

Other differences between regions, such as the level of landscape fragmentation or the difference in flowering times, could also generate differences in the level of gene flow (Rovere & Chalcoff, 2010). Climate has been shown to affect the flowering period of *E*. *coccineum*, for which the flowering period begins later (in November–December) in populations located at high latitudes and altitudes compared with the rest of the distribution (where it starts in September–October; Chalcoff, 2008; Hoffmann, 1997; Rovere & Chalcoff, 2010). Limited gene flow between populations presenting differences in flowering phenology could also lead to the observed pattern of divergence between and within genetic groups by limiting exchange between close populations located at contrasting altitudes. In *Protea rapens*, a widespread Proteaceae from the Cape Floristic Region in South Africa, a genetic split between eastern and western populations has been reported (Prunier et al., 2017). Differences in the yearly distribution of rainfall between the east and the west were proposed as drivers of population differentiation and restricted geneflow, as these differences have a direct influence on the flowering period of *P*. *rapens* (Heelemann et al., 2008).

### 4.3 Signatures of local adaptation in *Embothrium coccineum*

One of our goals was to differentiate between the effects of local adaptation and isolation by environment (IBE) and those of neutral processes, such as isolation by distance (IBD) or co‐ancestry (IBA, linked to the species glacial history) on the genetic differentiation in *E*. *coccineum*. Our results confirmed that the genetic variation is, at least in part, driven by the adaptation to strong environmental gradients characteristic of the species' distribution range (Daniels & Veblen, 2000; Souto & Premoli, 2007; Souto & Smouse, 2014; Souto et al., 2009). Indeed, the redundancy analysis (RDA) indicated that IBE was a significant driver of population structure that explained the largest amount of among‐population variation, even after controlling for IBD and IBA. These results were corroborated by the much higher divergence detected between the Northern and Central‐Southern genetic groups of the NJ tree based on the genetic distance calculated only for outlier loci (putatively under selection) compared with the genetic distance calculated for all the loci genotyped.

Our gradientForest analysis identified the precipitation of the driest month as the most critical explanatory environmental variable for the pattern of genetic variation in *E*. *coccineum*. The level of precipitation of the driest month discriminated the Mediterranean biome to the north and the Patagonian steppe to the east (both with very low summer precipitation) from the temperate biome inhabited by *E*. *coccineum* (Escobar et al., 2006). Water stress is known to be an active driver of adaptive divergence in terrestrial plants and the level of precipitation is possibly the environmental variable with the highest impact on their survival (Allen et al., 2010; Engelbrecht et al., 2007). In addition, we detected other limiting factors influencing adaptive genetic variation in *E*. *coccineum* such as mean temperature of the wettest quarter and mean temperature of the driest quarter as well as elevation that together could act as drivers of natural selection among populations (Varas‐Myrik et al., 2022). The covariation between these environmental variables and latitude was supported by the fact that four of the putative outlier loci had the highest correlation with environmental factors. The allelic frequency of these loci thus changed gradually with latitude. These results emphasized that genetic differentiation in *E*. *coccineum* could be mainly associated with environmental differences among populations related to summer rainfall and winter temperature. Other studies have also found that climate and in particular precipitation lead to genetic outliers in plants and underpin the development of drought‐adaptive phenotypic response (i.e., physiological and morphological traits) to the environment (Galliart et al., 2020; Gates et al., 2019).

Differences in morphology associated with environmental gradients are already known in *E*. *coccineum* (Chalcoff, 2008; Souto & Premoli, 2007) and further support the idea of possible local adaptation (current study). These studies also found that access to water strongly shaped the distribution of ecotypes in each region; where individuals grew as short trees of 0.5–2.5 m in height with small leaves in the driest climate of the northern region, while the same species reached more than 10 m height and developed longer leaves in the wet temperate forests of the central region (Souto & Smouse, 2014; Souto et al., 2009). Plants in areas with steady and high rainfall are less affected by heat and droughts, which may favor the production of larger, broader leaves to maximize the photosynthetic area at low risks of overheating and desiccation (Givnish, 1987; Hallik et al., 2009). In fact, the reduction in leaf size has been proposed as a key strategy to withstand water deficit (Baldocchi & Xu, 2007; Peguero‐Pina et al., 2014). In dry environments, such as those found in the northern distribution of *E*. *coccineum*, leaves with low Specific Leaf Area (SLA) are more resistant to wilting (Cunningham et al., 1999) and last longer than leaves with high SLA, which helps the plant spare resources (Reich et al., 2003). Additionally, small, narrow leaves have less surface area, which reduces water loss, and their thinner boundary layers provide higher heat shedding and compensate for the absence of transpirational cooling (Fonseca et al., 2000; Yates et al., 2010). In *Quercus faginea*, a reduction in leaf size has been proposed as critical among Mediterranean oaks to withstand the water deficit characteristic of the region (Baldocchi & Xu, 2007, Peguero‐Pina et al., 2014).

## 5 CONCLUSION AND PROSPECTS

Known patterns of morphological variations coupled with new results of genetic diversity and structure obtained during the present study revealed a long history of geographic isolation and local adaptation in *E*. *coccineum* from South American temperate forests. Here, we observed a strong genetic structure in *E*. *coccineum* in part linked to local adaptation, especially related to the access to water during the driest months. Our results support previous findings that reported the existence of distinct ecotypes along the species' natural distribution. We further identified and quantified the environmental variables linked to genetic population structuring, which can aid management decisions, conservation, restoration, or reforestation purposes. For example, knowledge on the association of genotypes with environmental conditions can become crucial when selecting proper seeds, especially in species with locally adapted populations, as it is the case in *E*. *coccineum*. Moreover, as the environment changes, nonlocal seed sources may also become a considerable source for restoration or reforestation purposes and can be selected according to their match to the novel environment, if this information is available. Therefore, seed transfer guidelines can benefit from data on factors structuring the landscape of genetic variation. Many conservation efforts rely on delineating distinct populations for management but often ignore the continuous nature of landscape variation and its potential relationship to local adaptation or other processes that lead to genotype–environment associations. Concerning land management decisions, the present results advocate for seed transfer guidelines that should be restricted to each ecological zone. The strong genetic structure also offers an opportunity to further explore local adaptation among groups and exploit these for agroforestry.

## AUTHOR CONTRIBUTIONS

**Francisco Sepúlveda‐Espinoza:** Conceptualization (equal); data curation (equal); formal analysis (lead); investigation (lead); methodology (lead); validation (lead); visualization (equal); writing – original draft (equal); writing – review and editing (equal). **Ariana Bertin‐Benavides:** Conceptualization (equal); data curation (equal); formal analysis (equal); funding acquisition (lead); investigation (lead); methodology (equal); project administration (lead); resources (lead); supervision (equal); validation (equal); writing – original draft (equal); writing – review and editing (equal). **Rodrigo Hasbún:** Formal analysis (supporting); investigation (supporting); methodology (equal); writing – original draft (equal); writing – review and editing (equal). **Óscar Toro‐Núñez:** Conceptualization (equal); formal analysis (equal); investigation (equal); methodology (equal); validation (equal); writing – review and editing (equal). **Antonio Varas‐Myrik:** Data curation (equal); formal analysis (equal); investigation (equal); methodology (equal); validation (equal); writing – review and editing (equal). **Diego Alarcón:** Formal analysis (equal); investigation (equal); methodology (equal); visualization (equal); writing – review and editing (equal). **Marie Guillemin:** Data curation (equal); formal analysis (lead); investigation (lead); methodology (equal); supervision (lead); validation (equal); writing – original draft (lead).

## CONFLICT OF INTEREST

On behalf of all authors, the corresponding author states that there is no conflict of interest.

### OPEN RESEARCH BADGES

This article has earned Open Data, Open Materials and Preregistered Research Design badges. Data, materials and the preregistered design and analysis plan are available at https://www.ncbi.nlm.nih.gov/sra/?term=PRJNA783610.

## Supporting information


Appendix S1.

## Data Availability

The authors declare that all the work presented in this manuscript was done according to the standards of the journal *Ecology and Evolution*. Raw GBS data used in this study are available in the ncbi‐SRA database at http://www.ncbi.nlm.nih.gov/sra/?term=PRJNA783610.
