# Supplementary material for: The impact of Pleistocene glaciations and environmental gradients on the genetic structure of Embothrium coccineum
Source: Ecol Evol. 2022 Nov 9;12(11):e9474. doi: 10.1002/ece3.9474 (PMC9646505; doi:10.1002/ece3.9474)
Supplement: Supplementary file 1 — Appendix S1. [file ECE3-12-e9474-s001.docx]

**Appendix S1**

**Table S****1: Environmental characteristics of each sampling location of *Embothrium coccineum.***

GPS coordinates are given for each sampling location. Environmental variables: bio 14, Precipitation of the driest month (mm); bio 8, Mean temperature of the wettest quarter (°C); Elv, Elevation (mamsl); bio 9, Mean temperature of the driest quarter (°C); bio 4, Temperature seasonality (°C); bio 3, Isothermality (%); bio 13, Precipitation of the wettest month (mm).

| **Locations** | **ID** | **Latitude** | **Longitude** | **bio 14** | **bio 8** | **Elv** | **bio 9** | **bio 4** | **bio 3** | **bio 13** |
| --- | --- | --- | --- | --- | --- | --- | --- | --- | --- | --- |
| Chillán | Ch | -36.908 | -71.504 | 23 | 3.9 | 1216 | 14.0 | 4.1 | 55 | 257 |
| Curacautín | Cu | -37.642 | -73.099 | 28 | 4.4 | 989 | 11.2 | 2.8 | 57 | 297 |
| Nahuelbuta | Nah | -38.455 | -71.734 | 53 | 5.5 | 797 | 14.7 | 3.7 | 56 | 390 |
| Puerto Montt | PM | -41.521 | -72.753 | 95 | 7.9 | 81 | 14.9 | 2.9 | 49 | 267 |
| Chiloe Norte | ChlN | -42.296 | -73.459 | 90 | 7.7 | 129 | 13.5 | 2.4 | 47 | 333 |
| Chiloe Sur | ChlS | -42.783 | -73.806 | 85 | 8.0 | 97 | 13.4 | 2.3 | 45 | 303 |
| Pumalín | Pu | -42.536 | -72.496 | 96 | 7.4 | 22 | 15.4 | 3.3 | 49 | 309 |
| Coyhaique | Coy | -46.505 | -73.066 | 98 | 4.4 | 175 | 9.9 | 3.2 | 44 | 181 |
| Chile Chico | ChCh | -46.649 | -72.362 | 35 | 2.6 | 428 | 11.8 | 3.7 | 44 | 101 |
| Torres del Paine | TP | -51.361 | -72.799 | 37 | 9.1 | 33 | 9.7 | 3.4 | 48 | 67 |

**
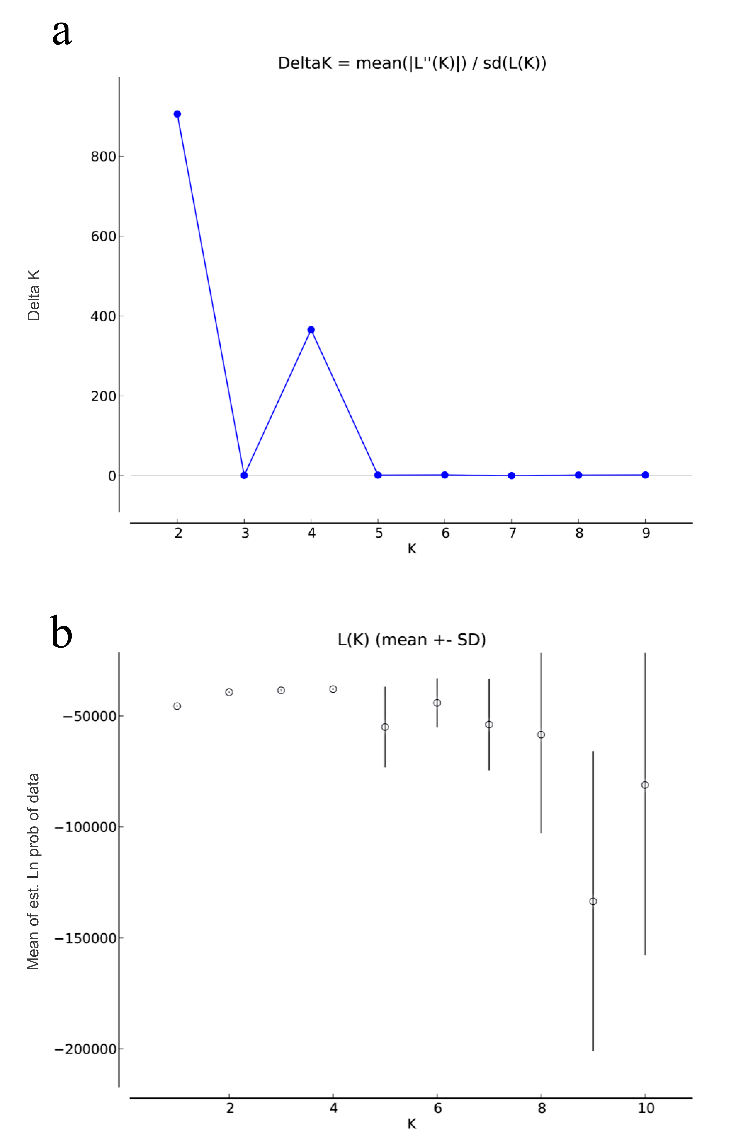
**

**Figure S1****:** Results of the model-based Bayesian clustering approach. Highest (a) ΔK and (b) LnP(D) show that the best K was K = 2.


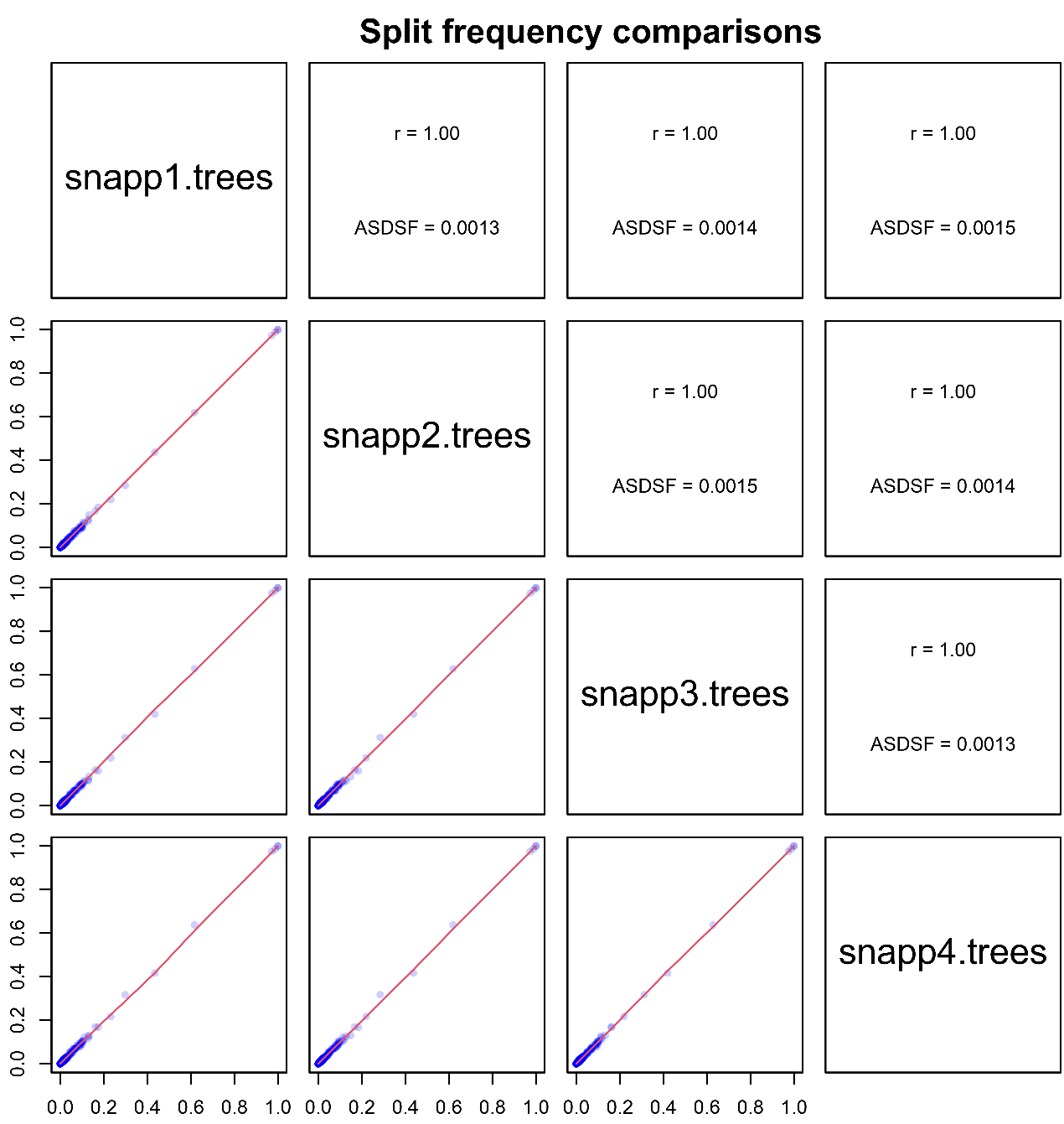


**Figure S2:** Convergence of split frequencies among each SNAPP run.


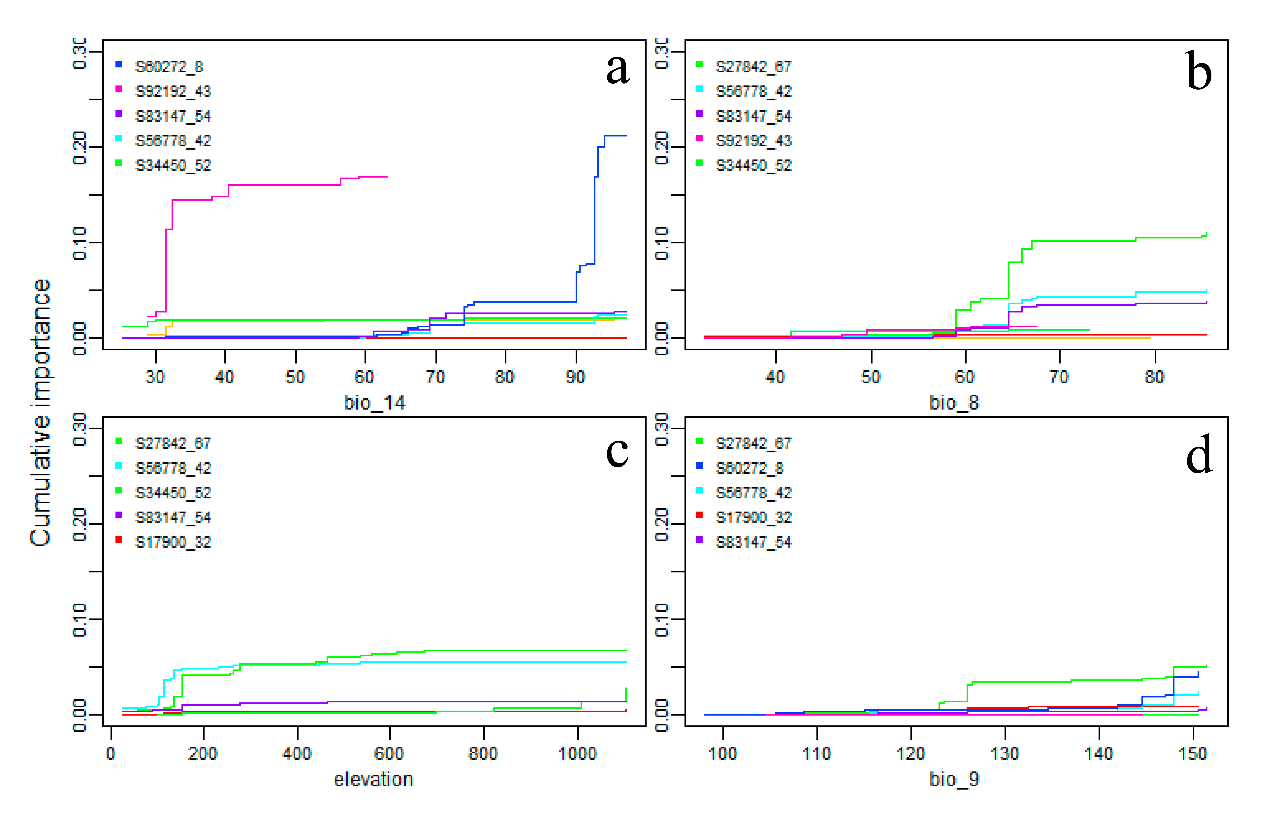


**Figure S3:** SNP-level compositional turnover functions from GF for 5 outliers loci along the gradient of: a) bio 14, Precipitation of the driest month (mm); b) bio 8, Mean temperature of the wettest quarter (°C); c) Elevation (masl); d) bio 9, Mean temperature of the driest quarter (°C).
